# Supplementary material for: Wide spectrum of neuronal and network phenotypes in human stem cell-derived excitatory neurons with Rett syndrome-associated MECP2 mutations
Source: Transl Psychiatry. 2022 Oct 18;12:450. doi: 10.1038/s41398-022-02216-1 (PMC9576700; doi:10.1038/s41398-022-02216-1)
Supplement: Supplementary file 1 — Supp Tables and Supp Fig Legends [file 41398_2022_2216_MOESM1_ESM.docx]

**Supplemental Tables and Figure Legends**

**Reagent Lists**

| **Individual** | **Cell**  **Type** | **Genotype** | **# of**  **Clones** | ***MECP2*^+/-^**  **Mutation Description** | **Reference** |
| --- | --- | --- | --- | --- | --- |
| CLT | iPSC | WT | 2 | Wild-type | This study |
|  |  | L124W | 2 | Patient c.371T>G; L124W missense mutation in methyl binding domain |  |
| d3-4 | iPSC | WT | 1 | Wild-type | Cheung et al, 2011 |
|  |  | NULL | 1 | Patient indel disrupting exons 3 & 4 |  |
| WIBR3 | ESC | WT | 1 | Wild-type | Li et al, 2013 |
|  |  | NULL | 1 | TALEN-edited deletion of exons 3 & 4, Clone #913 from Yun Li. |  |
| PGPC14 | iPSC | WT | 1 | Wild-type | Hildebrandt et al, 2019 |
|  |  | NULL | 1 | CRISPR/Cas9-edited 1 bp insertion (frameshift, premature stop codon) | This study |

Table S1. Description of iPSC/ESC lines.

| **Application** | **Target** | **FWD (5’-3’)** | **REV (5’-3-)** | **Source** |
| --- | --- | --- | --- | --- |
| XCI | *AR* | CGTGCGCGAAGTGAT  CCAGA | GTTTCTTTGCTGCTGC  CTGGGGCTAGT | PMID: 21372149 |
| cDNA sequencing | *MECP2* | GCTCCATAAAAATACAGACTCAC | TTTGGGCTTCTTAGGTGGTTT | This study |
| CRISPR sgRNA | *MECP2* | AGAAGCTTCCGGCACAGCCG | | This study |

Table S2. Oligonucleotide sequences.

| **Antibody** | **Species** | **Dilution** | | **Source** | **Cat #** |
| --- | --- | --- | --- | --- | --- |
|  |  | **ICC** | **Wes** |  |  |
| Anti-α-SMA | Mouse | 1:200 |  | Invitrogen | 18-0106 |
| Anti-β-actin | Mouse |  | 1:500 | Sigma | A5441 |
| Anti-β-III tubulin | Mouse | 1:200 |  | Chemicon | MAB1637 |
| Anti-AFP | Mouse | 1:200 |  | R&D | MAB1368 |
| Anti-GFP | Chicken | 1:1000 |  | Thermo Fisher | A10262 |
| Anti-HOMER1 | Guinea pig | 1:500 |  | Synaptic Systems | 160004 |
| Anti-MAP2 | Mouse | 1:1000 |  | Sigma | M1406 |
| Anti-MAP2 | Guinea pig | 1:1000 |  | Synaptic Systems | 188004 |
| Anti-MECP2 | Rabbit | 1:1000 | 1:50 | Cell Signalling | 3456 (D4F3) XP |
| Anti-Nanog | Rabbit | 1:200 |  | Cell Signalling | 4903P |
| Anti-OCT4 | Rabbit | 1:200 |  | Abcam | ab19857 |
| Anti-SSEA4 | Mouse | 1:100 |  | Invitrogen | 41-4000 |
| Anti-SYN1 | Rabbit | 1:200 |  | Millipore | AB1543P |
| Anti-TRA-1-60 | Mouse | 1:100 |  | Invitrogen | 41-1000 |

Table S3. Primary antibodies.

**SUPPLEMENTAL FIGURE CAPTIONS**

*Figure S1. Generation of MECP2 L124W and PGPC14 MECP2 null iPSCs and Ngn2 excitatory cortical neurons.*

**A.** G-band karyotyping of two CLT WT (C4 and C17) and two CLT L124W (C1 and C2) female iPSC lines (46XX).

**B.** Representative images of iPSC colonies from two CLT WT (C4 and C17) and two CLT L124W (C1 and C2) lines stained with pluripotency-associated nuclear markers OCT4 and NANOG (red) and surface markers SSEA4 and TRA-1-60 (green). Scale bar = 50 μm.

**C.** Representative images of spontaneous 3-germ layer embryoid body differentiations from two CLT WT (C4 and C17) and two CLT L124W (C1 and C2) iPSC lines stained with mesoderm marker SMA, ectoderm marker β-III-tubulin and endoderm marker AFP (green). Scale bar = 50 μm.

**D.** Pluritest clustering of two CLT WT (C4 and C17) and two CLT L124W (C1 and C2) iPSC colonies based on gene expression profile comparison to established iPSC lines.

**E.** Schematic of induced *Ngn2* neuronal differentiation protocol from iPSCs/ESCs.

**F.** G-band karyotyping of PGPC14 null iPSC line (46XX).

**G.** Representative images of iPSC colonies from PGPC14 null line stained with pluripotency-associated nuclear markers OCT4 and NANOG (green) and surface markers SSEA4 and TRA-1-60 (magenta). Scale bar = 50 μm.

**H.** Sanger sequencing of cDNA for the 1 bp insertion frameshift mutation (p.Val74CysfsTer16) from restricted expression of the active X chromosome CRISPR/Cas9 gene edited PGPC14 iPSCs and the sgRNA used.

**I.** Representative images of 6 week old PGPC14 WT and null *Ngn2* neurons co-cultured on mouse astrocytes stained with MECP2 (green) and MAP2 (magenta). Scale bar = 50 μm.

**J.** Western blot of MECP2 protein in 6 week old PGPC14 WT and null *Ngn2* neurons, compared to 4 week old d3-4 WT and null NPC-derived neurons.

*Figure S2. Alterations in the parameters of action potentials in RTT Ngn2 neurons. .*

**A-E.** Scatter plots showing all data points for the threshold, amplitude, rise time, half-duration and decay time of evoked action potentials in 4-5 week-old *Ngn2* neurons of WT (n = 66, 42, and 99, respectively) and RTT from d3-4 (n =58), WIBR (n = 55), CLT (n = 100). The graphs also display average parameters of action potentials. The data are shown as mean +/- SEM. Liquid junction potential was not corrected for the action potential threshold as plotted. Statistical significance was evaluated by Student’s *t*-test or Mann Whitney test, as appropriate. *p < 0.05, ***p < 0.001.

*Figure S3. Branching alterations in RTT Ngn2 neurons.*

**A.** Quantification of 6 week old WT and RTT *Ngn2* neurons co-cultured with mouse astrocytes, for dendrite branching order (manual counting). Mean +/- SEM, * p < 0.05.

*Figure S4. MEA properties in RTT Ngn2 neurons.*

**A.** Representative images of 5-7 week old cultures on MEA plates. Scale bar = 100 μm.

**B.** Number of active electrodes for isogenic WT and RTT *Ngn2* neurons over 3 to 7 weeks of individual replicate plates (number above indicate Plate ID). 6 replicate plates of WIBR3, 4 replicate plates of PGPC14 and 6 replicate plates of CLT showing all 4 isogenic lines, with 2-6 active wells analyzed per genotype per plate. Some plates include extra recordings at half-hour intervals, but only the hourly recordings acquired from all plates were used in analyses. Mean +/- SEM shown.

**C.** Mean firing rate of 10 week old WT and *MECP2* null *Ngn2* neurons treated with CNQX.

**D-F.** Weighted mean firing rate, network burst frequency and duration for isogenic WT and null *Ngn2* neurons over 3 to 7 weeks of individual replicate plates (number above indicates Plate ID). 6 replicate plates of WIBR3, 4 replicate plates of PGPC14 and 6 replicate plates of CLT, with 2-6 active wells per genotype per plate. Mean with SEM. 2 lines of isogenic WT and 2 lines of CLT L124W data are shown.

*Figure S5. Network analysis in RTT Ngn2 neurons.*

**A.** Distribution of bursting frequencies across 6 WIBR3 plates, 4 PGPC14 plates, and 5 L124W CLT plates. Medians in each case are plotted as blue dots. Because only electrodes with detected bursts are plotted, the number of samples increases from week 2 to 7 (noted above each histogram). Statistical significance was evaluated by Mann Whitney test. ***p < 0.001.

**B.** Control simulation to understand the effect of connectivity (vertical axis) and subthreshold adaptation (horizontal axis). Each point in the heatmap represents the measured burst frequency (colorbar) at one combination of connection probability and subthreshold adaptation.
