# Supplementary figures and images for: Wide spectrum of neuronal and network phenotypes in human stem cell-derived excitatory neurons with Rett syndrome-associated MECP2 mutations
Source: Transl Psychiatry. 2022 Oct 18;12:450. doi: 10.1038/s41398-022-02216-1 (PMC9576700; doi:10.1038/s41398-022-02216-1)

Figure S1

A

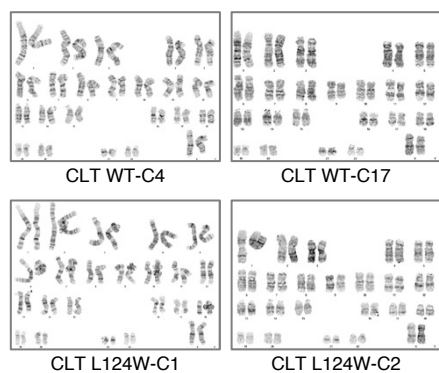

B

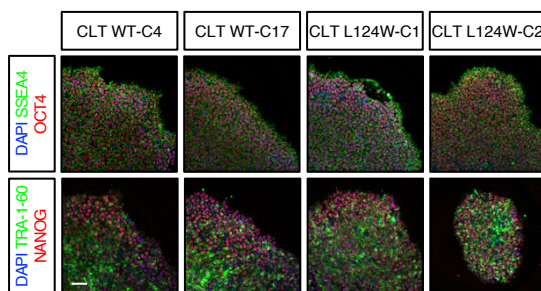

C

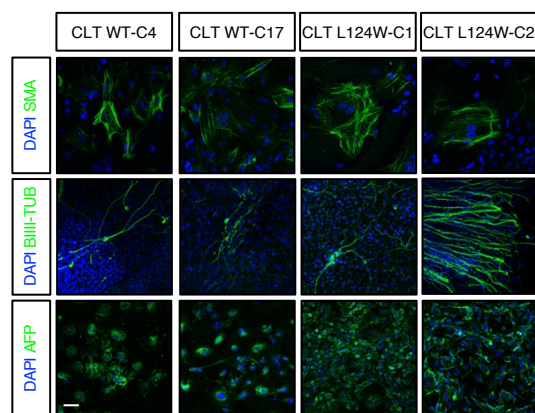

D

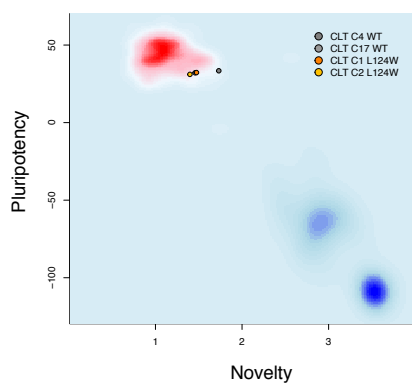

E

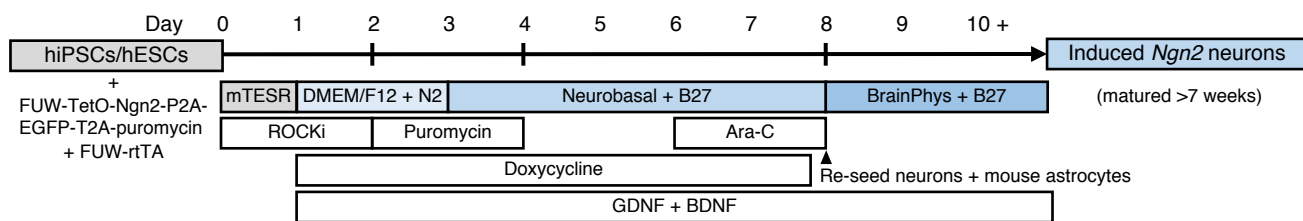

F

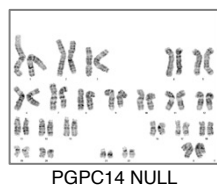

G

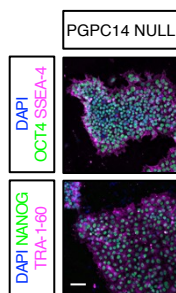

H

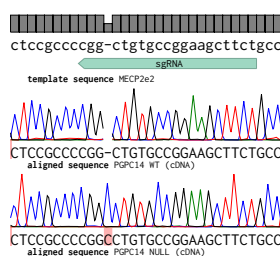

I

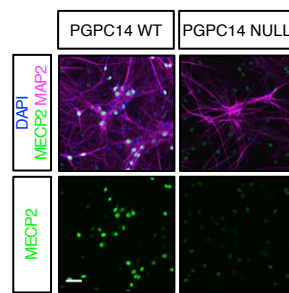

J

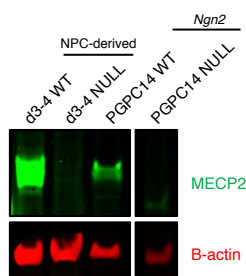

Figure S2

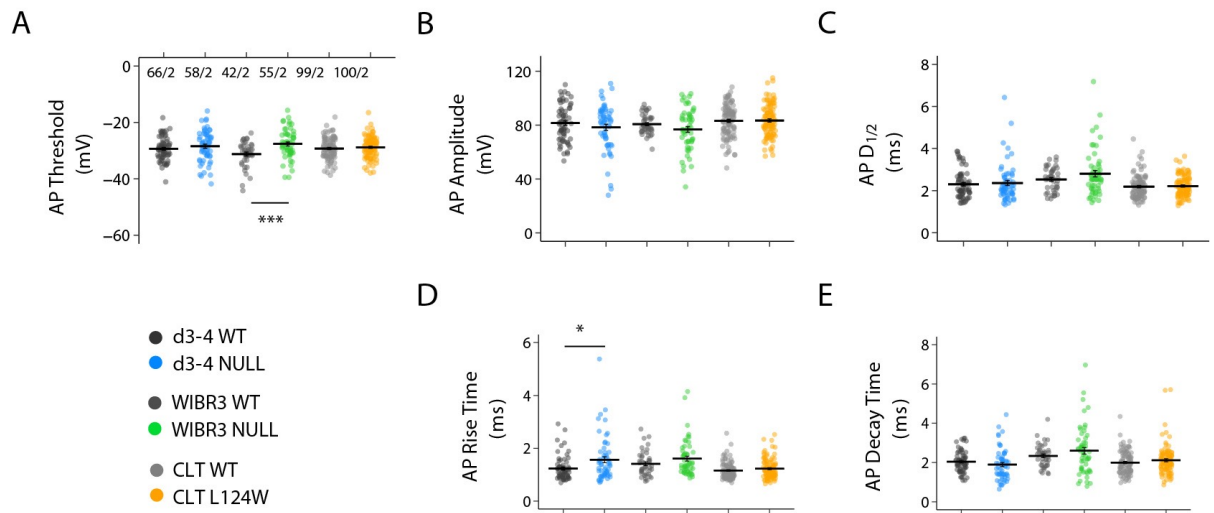

Figure S3

A

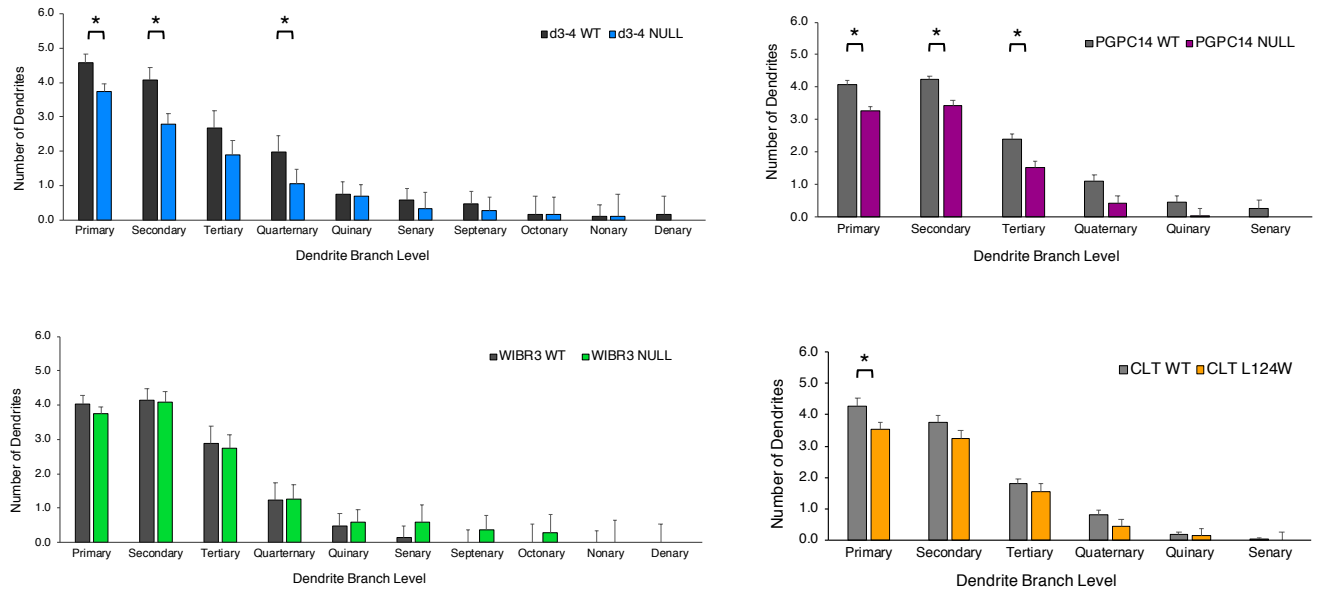

Figure S4

A

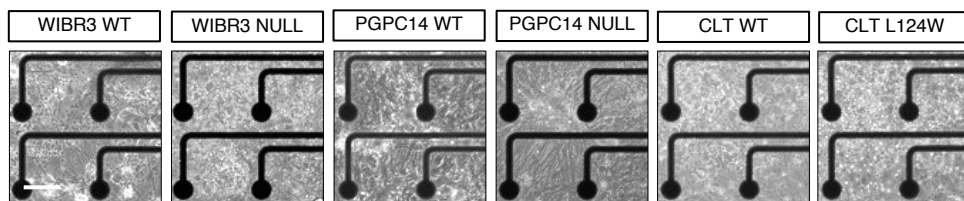

C

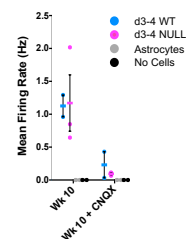

B

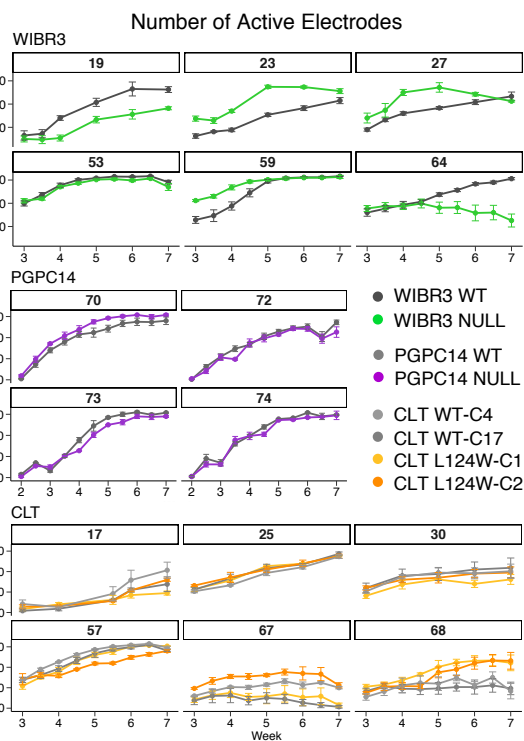

D

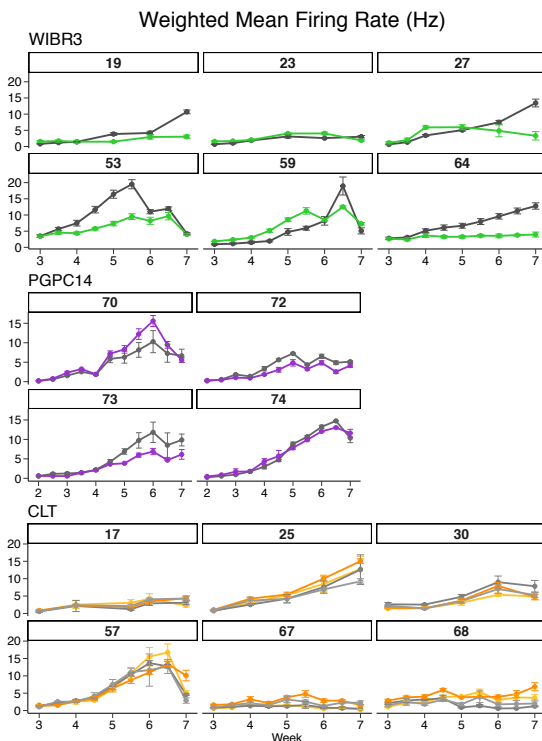

E

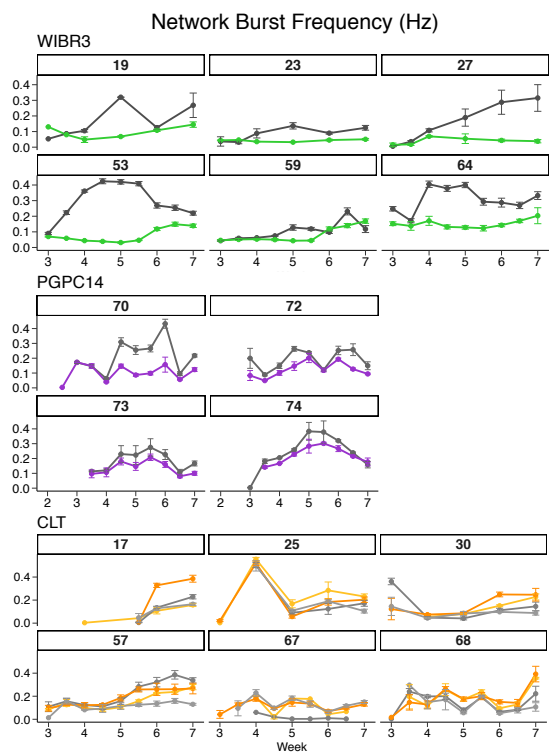

F

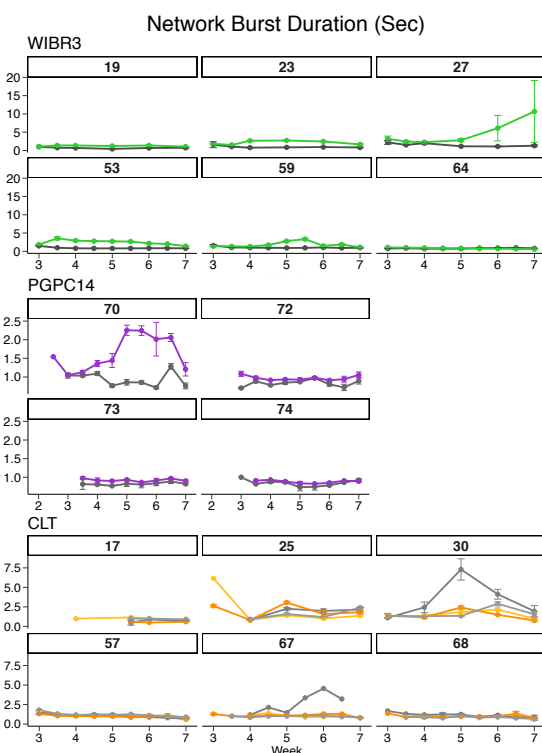

Figure S5

A

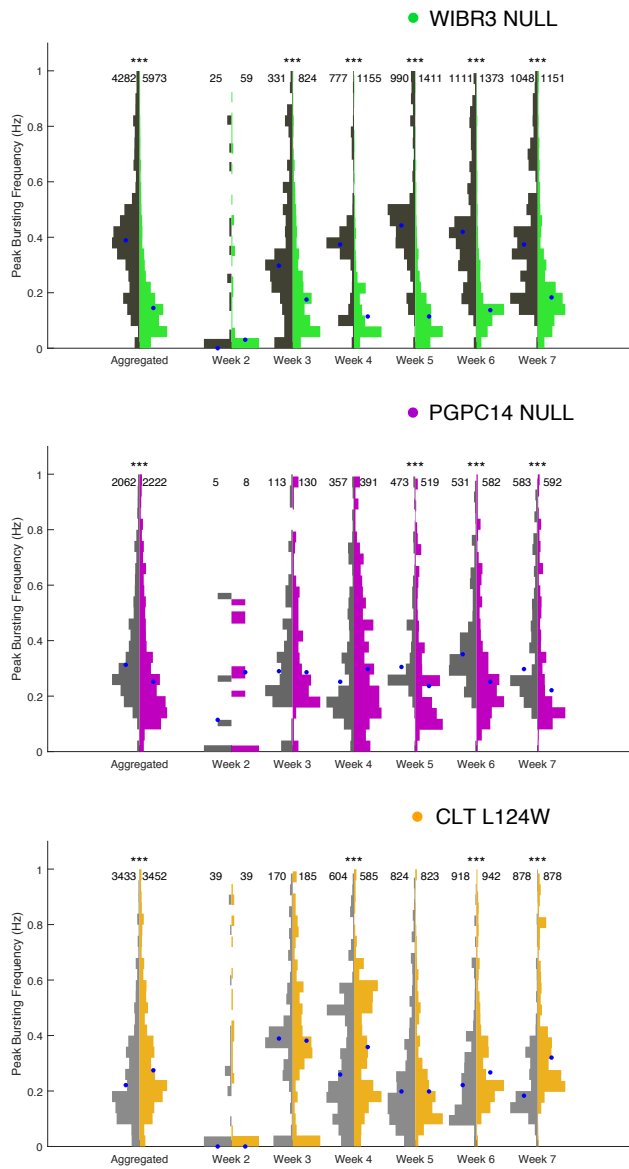

B

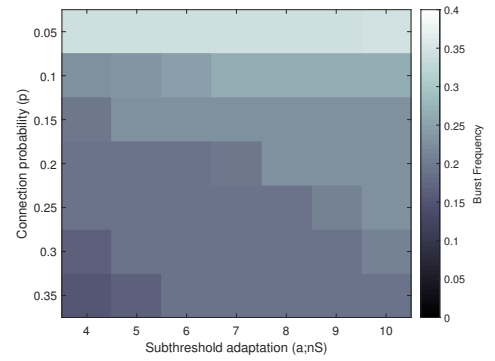

Supplement: Supplementary file 2 — Supp Figures [file 41398_2022_2216_MOESM2_ESM.pdf]
